# Viable Allogeneic Mitochondria Transplantation Improves Gas Exchange and Alveolar-Capillary Permeability in Rats with Endotoxin-Induced Acute Lung Injuries

**DOI:** 10.7150/ijms.73151

**Published:** 2022-06-06

**Authors:** Yu-Li Pang, Shi-Yuan Fang, Tzu-Ting Cheng, Chien-Chi Huang, Ming-Wei Lin, Chen-Fuh Lam, Kuen-Bao Chen

**Affiliations:** 1 Department of Anesthesiology, Chi Mei Medical Center, Tainan 710, Taiwan.; 2 Department of Anesthesiology, National Cheng Kung University Hospital, College of Medicine, National Cheng Kung University, Tainan 704, Taiwan.; 3 Department of Anesthesiology, E-Da Hospital and E-Da Cancer Hospital, Kaohsiung 824, Taiwan.; 4 Department of Medical Research, E-Da Hospital and E-Da Cancer Hospital, Kaohsiung 824, Taiwan.; 5 School of Medicine, I-Shou University College of Medicine, Kaohsiung 824, Taiwan.; 6 Department of Anesthesiology, China Medical University Hospital, Taichung 404, Taiwan.

**Keywords:** ATP, ARDS, endothelial function, hyaline membrane, pulmonary artery

## Abstract

**Background:** Acute lung injuries (ALI) cause disruption of the alveolar-capillary barrier and is the leading cause of death in critically ill patients. This study tested the hypothesis that the administration of freshly isolated viable allogeneic mitochondria can prevent alveolar-capillary barrier injuries at the endothelial level, as mitochondrial dysfunction of the pulmonary endothelium is a critical aspect of ALI progression.

**Methods:** ALI was induced by intratracheal lipopolysaccharide instillation (LPS, 1mg/kg) in anesthetized rats. Mitochondria (100 µg) were isolated from the freshly harvested soleus muscles of naïve rats and stained with a green fluorescence MitoTracker™ dyne. A mitochondria or placebo solution was randomly administered into the jugular veins of the rats at 2 h and 4 h after ALI induction. An arterial blood gas analysis was done 20 h later. The animals were then sacrificed and lung tissues were harvested for analysis.

**Results:** An IVIS Spectrum imaging system was used to obtain *ex vivo* heart-lung block images and track the enhancement of MitoTracker™ fluorescence in the lungs. Mitochondria transplantation significantly improved arterial oxygen contents (PaO_2_ and SaO_2_) and reduced CO_2_ tension in rats with ALI. Animals with mitochondrial transplants had significantly higher ATP concentrations in their lung tissues. Allogeneic mitochondria transplantation preserved alveolar-capillary barrier function, as shown by a reduction in protein levels in the bronchoalveolar lavage fluid and decreased extravasated Evans blue dyne and hemoglobin content in lung tissues. In addition, relaxation responses to acetylcholine and eNOS expression were potentiated in injured pulmonary arteries and inflammatory cells infiltration into lung tissue was reduced following mitochondrial transplantation.

**Conclusions:** Transplantation of viable mitochondria protects the integrity of endothelial lining of the alveolar-capillary barrier, thereby improving gas exchange during the acute stages of endotoxin-induced ALI. However, the long-term effects of mitochondrial transplantation on pulmonary function recovery after ALI requires further investigation.

## Introduction

Acute lung injury (ALI) and acute respiratory distress syndrome (ARDS) represent the most common causes of intensive care unit (ICU) admission, need for ventilatory support, and death in critically ill patients with a high mortality rate of up to 35-45% 1. In severe ALI, intense pro-inflammatory responses and leukocyte infiltration into the lung tissue destroys the integrity of the alveolar epithelial and pulmonary artery endothelial layers, leading to the breakdown of the alveolar-epithelial barrier. This promotes the formation of protein-rich pulmonary edema and impairs blood-gas exchange 2-4. Since the pulmonary vascular endothelium is the key modulator of ALI progression, pharmaceutic- and cell-based endothelial-targeting therapies have become emerging candidate therapies for ALI/ARDS in the past two decades 5, 6.

Under normal conditions, the pulmonary vascular endothelium inhibits the majority of inflammatory and coagulation responses in the lung; but in ALI, these endothelial cells are activated and contribute to the pro-inflammatory and pro-coagulant responses 7. Mitochondria serve as the most important sensors in regulating intra- and inter-cellular communications for the maintenance of healthy cellular homeostasis. In order to maintain homeostasis, mitochondria are also extremely sensitive to environmental stressors 8. The cellular environment can become toxic or allostatic under excessive physical stress, and defects in mitochondrial electron transport, intracellular Ca^2+^ homeostasis, ion transport, mitochondrial biogenesis, cellular metabolism, redox regulation, and mitochondrial quality control result in mitochondrial dysfunction 8, 9. Recent evidence suggests that impaired mitochondrial homeostasis in pulmonary epithelial and vascular endothelial cells contributes to the progress of ALI 10, 11 and the loss of mitochondrial genomic integrity is the crucial initial step that drives lung injury propagation during ALI 12. Since viable exogeneous mitochondria transplantation has been shown therapeutic potential in various pre-clinical ischemia-reperfusion injury models 13-15 and in pediatric patients with myocardial dysfunction 16, we conducted the first pre-clinical study to test the hypothesis that the administration of freshly-isolated viable allogeneic mitochondria can reduce endothelial injury at the alveolar-capillary barrier and improve gas exchange in an experimental model of endotoxin-induced ALI.

## Methods

### Rat model of acute lung injury

All experimental procedure were performed in accordance with the ARRIVE Guideline and the guidelines for animal research issued by the E-Da Hospital and I-Shou University. The study was approved by the Institutional Animal Care and Use Committee of the E-Da Hospital, Kaohsiung, Taiwan (IACUC approval number 109024). Adult male Sprague-Dawley rats were anesthetized using intraperitoneal Zoletil cocktail anesthetic injections (30 mg/kg). Following a mini-open incision, a placebo solution (0.9% saline, 0.5 ml) or Escherichia coli lipopolysaccharide (LPS, 1 mg/kg in 0.5 ml of saline; Sigma-Aldrich; Merck KGaA, Darmstadt, Germany) was instilled through the glottis with a catheter for sham operations and ALI induction respectively 17.

### Isolation and administration of mitochondria

Mitochondria were freshly isolated from healthy naïve rats within 1h before delivery. Rats were asphyxiated with CO_2_ and bilateral soleus muscles were harvested. A commercially available mitochondria isolation kit (Thermo Scientific, Waltham, MA) was used to isolate viable mitochondria 18. The freshly isolated soleus muscles were briefly washed with a phosphate buffer solution (PBS, containing 0.1 M sodium phosphate and 0.15 M sodium chloride at pH 7.2) then incubated with a tryptase solution (0.3 mg/ml) for 3 minutes. The muscles were then homogenized using a grinder and the resulting tissue samples were centrifuged at 11,752 g for 3 minutes at 4 ºC. After three cycles of suspension and centrifugation, the mitochondrial pellets were collected and the cytosolic components were discarded using mitochondria isolation reagents (A to C solutions).

### Allocation of Experimental Groups and Study Protocol

Animals were randomly allocated to sham (0.9% saline) or ALI group (LPS). Rats in the ALI group were then randomly re-assigned to either the control or mitochondrial group. The control group received intravenous PBS (200 μL) and the mitochondria group received skeletal muscle-derived mitochondria in PBS (100 μg in 200 μL PBS). Solutions in both groups were delivered via the jugular veins. Each animal in the control or mitochondrial group received the treatment solution at 2 h and 4 h after intratracheal instillation of LPS and they recovered from anesthesia under a warm blanket. A total of 11 rats received the sham operation, 23 rats received LPS treatment (including 10 controls and 13 mitochondrial transplantation), and 6 rats were served as naïve animals for tissue harvesting and *ex vivo* image scanning. The experimental animals were sacrificed under deep anesthesia 24 h after instillation of saline or LPS to collect blood samples and lung tissues for analysis.

### Identification of the Distribution of Exogenous Mitochondria following systemic delivery

In some experiments, the isolated mitochondria were stained with a green MitoTracker™ fluorescence dyne (catalog #M7514, Invitrogen™, Carlsbad, CA) for the *ex vivo* imaging under an *in vivo* imaging system (IVIS, Caliper IVIS Spectrum System, PerkinElmer, Waltham, MA). Heart and lungs were harvested *en bloc* at 30 min after intravenous delivery of MitoTracker fluorescence-labelled mitochondria and scanned under the 3-dimensional tomography imaging system.

### Artery blood gas analysis (ABG)

Arterial blood was drawn from the femoral artery of anesthetized rats under room air conditions. Oxygen saturation (SaO_2_), partial pressure of oxygen (PaO_2_) and CO_2_ (PaCO_2_), and other ABG parameters in the arterial blood were analyzed using a blood gas analyzer (ABL 520, Radiometer, Copenhagen, Denmark).

### Measurement of vascular reactivity

Pulmonary artery rings (approximately 2 mm long) were freshly isolated and mounted in organ chambers containing 25 ml of Krebs solution. The chambers were maintained at 37 ºC and aerated continuously with 94% O_2_/6% CO_2_. Changes in isometric force were recorded continuously using an isometric force-displacement transducer (Grass IT30; Grass Instrument, West Warwick, RI). Each ring was gradually stretched to 2.0 g. After a 45-min equilibration period, the rings were induced to contract using KCl (40 mM) and slow phenylephrine titration (PE, 10^-9^ to 10^-5^ M). Concentration-response curves were charted by recording acetylcholine (10^-9^ to 10^-5^ M; Sigma-Aldrich, Burlington, MA) during contraction to a median PE effective concentration (EC50).

### Assessment of pulmonary capillary permeability and lung wet-to-dry ratio

The permeability of alveolar-capillary barrier was assessed by measuring protein content in the bronchoalveolar lavage fluid (BALF), the Evans blue assay, and lung wet-to-dry ratio (LWDR) 19. The trachea in the lab animals were dissected and lungs were lavaged with 6 ml/kg chilled PBS. The recovered lavage fluid was analyzed for total protein content using a protein assay kit (Bio-Rad Laboratories, Hercules, CA). Evans blue dye (2% v/v, 20 mg/kg, Sigma Aldrich) was administered via the jugular vein at 30 min before euthanization and lung tissue was perfused free of blood with normal saline. Evans blue was extracted from the freshly isolated lung tissues by incubating in 99.5% formamide (4 ml/g tissue; Sigma Aldrich) for 48h and the tissue concentrations were determined using spectrophotometry at the wavelength of 620 nm. To assess lung water content, the left lung was excised and weighed immediately. Lung tissues were dried in an oven at 60 °C for 24 h and reweighed. LWDR was obtained by dividing the mass of the initial specimen by the mass of the dried specimen.

### Measurement of lung hemoglobin content and myeloperoxidase activity assay

The hemoglobin colorimetric assay was performed using a hemoglobin colorimetric assay kit (catalog #700540, Cayman Chemical, Ann Arbor, MI). Lungs were excised and homogenized in 5 ml PBS solutions (pH 7.4, with 0.16 mg/ml heparin, per gram weight of tissue). Homogenized lung tissues were centrifuged at 10,000 g for 15 minutes at 4 °C. After the supernatant was removed, the tissue samples were added to assay wells and incubated at room temperature for 15 minutes. The absorbance at 560-590 nm was read and analyzed. The enzymatic activity of myeloperoxidase (MPO) in the homogenized lungs was measured by a commercially available MPO assay kit (Cell Biolabs Inc., San Diego, CA).

### Measurement of Tissue adenosine triphosphate (ATP)

Tissue ATP concentrations were analyzed using an ATP assay kit (catalog #ab83355, Abcam, Cambridge, MA). The right lungs of lab animals were excised and homogenized in the ATP assay buffer. The homogenized tissue was centrifuged at 13,000 g for 5 minutes at 4 °C. Insoluble components were removed and the solution was deproteinized using a sample preparation kit. The supernatant was then collected for ATP colorimetric assay.

### Western Blot

Pulmonary artery trunk and lung tissues were minced and homogenized in lysis buffer. Equal amounts of proteins (100 mg) were loaded into polyacrylamide gels and transferred to nitrocellulose membranes using the wet transferring method. The membranes were incubated overnight at 4 °C with primary antibodies (endothelial nitric oxide synthase (eNOS), phosphorylated eNOS-S1177, CD11b and iNOS) in 1:1000 dilutions. All primary antibodies were purchased from BD Biosciences (New Jersey, USA), except for CD11b which was obtained from Abcam (Cambridge, MA). After washing with PBS, the membranes were incubated with horseradish peroxidase-linked secondary antibodies for 1 hour at room temperature. Bands were visualized using enhanced chemiluminescence and quantified with scanning densitometry (the ImageJ; 1.48v, National Institutes of Health, Bethesda, Md).

### Histologic and immunostaining examinations

Lung tissues were immersed in 10% buffered formal saline and fixed for 24 h. Biopsies were then processed through increasing grades of alcohol and embedded in paraffin wax. Hematoxylin and eosin (H&E)-stained lung sections were examined under a light microscope and photographed. Lung injury was assessed by an investigator blinded to the treatment groups on a scale of 0-2 for each of the following criteria: (1) infiltration of neutrophils in the alveolar and interstitial space, (2) amount of hyaline membrane and proteinaceous debris, (3) degree of lung hemorrhage and (4) extent of parenchymal consolidation 20.

### Statistical analysis

All data sets were tested for the normality assumption using the Shapiro-Wilk test before statistical procedures. The values of continuous variables were compared using the Mann-Whitney U test. Two-way repeated measures (RM) ANOVA was used to compare differences in vasomotor reactivity between the control and mitochondrial transplantation groups at the different vasoactive drug concentrations. A Dunn's post hoc procedure was used for multiple comparisons. Results are presented as the median and interquartile range (IQR). Statistical significance was accepted at a level of *P*< 0.05. All of the statistical analyses were performed using the SigmaPlot 14.0 (Systat Software Inc., San Jose, CA).

## Results

### Uptake of allogenic mitochondria following intravenous administration

**Figure 1:**
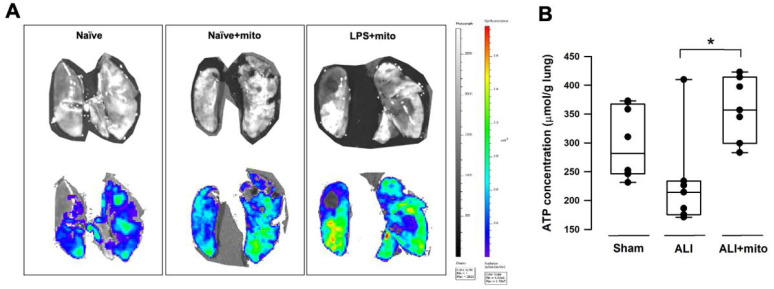
**(A)** Representative images acquired by an *in vivo* imaging system (IVIS) (Caliper IVIS Spectrum System). Mitochondria were freshly isolated from the soleus muscles of naïve rats and were labeled with a green fluorescence dyne (MitoTracker™ Green, Invitrogen™) at 30 min before intravenous administration. Upper panels were photographs taken under normal light and the lower panels were the fluorescence photographs taken under IVIS. Lower MitoTracker™ fluorescence densities (blue to green) were found in the lungs of naïve rats with or without mitochondria. Fluorescence densities were significantly enhanced in the lungs of rats treated with lipopolysaccharide (LPS) (yellow to red). Images were taken from 3 different animals in each group. **(B)** Tissue ATP concentration in the lungs of animals with ALI was restored following transplantation of freshly-isolated allogeneic mitochondria. Results are presented as box-and-whisker plots, in which the horizontal lines of color boxes indicate the 75^th^ percentile, median and 25^th^ percentile of the distribution, and the upper and lower whiskers indicate the maximal and minimal values. Data were analyzed using the Rank Sum Test, *P=0.009, n=8 different animals in each group.

Compared with naïve animals, MitoTracker™ Green fluorescence densities were better enhanced in the *ex vivo* lung lobes of rats with ALI that received skeletal muscle-derived allogeneic mitochondria transplantation (Figure 1A), indicating that exogenous mitochondria in the pulmonary circulation are retained following intravenous administration. Tissue ATP concentration in the lung homogenates of animals with ALI was restored following allogeneic mitochondrial transplantation (Figure 1B).

### Transplantation of mitochondria improves gas exchange and preserves function of alveolar-capillary barrier

**Figure 2:**
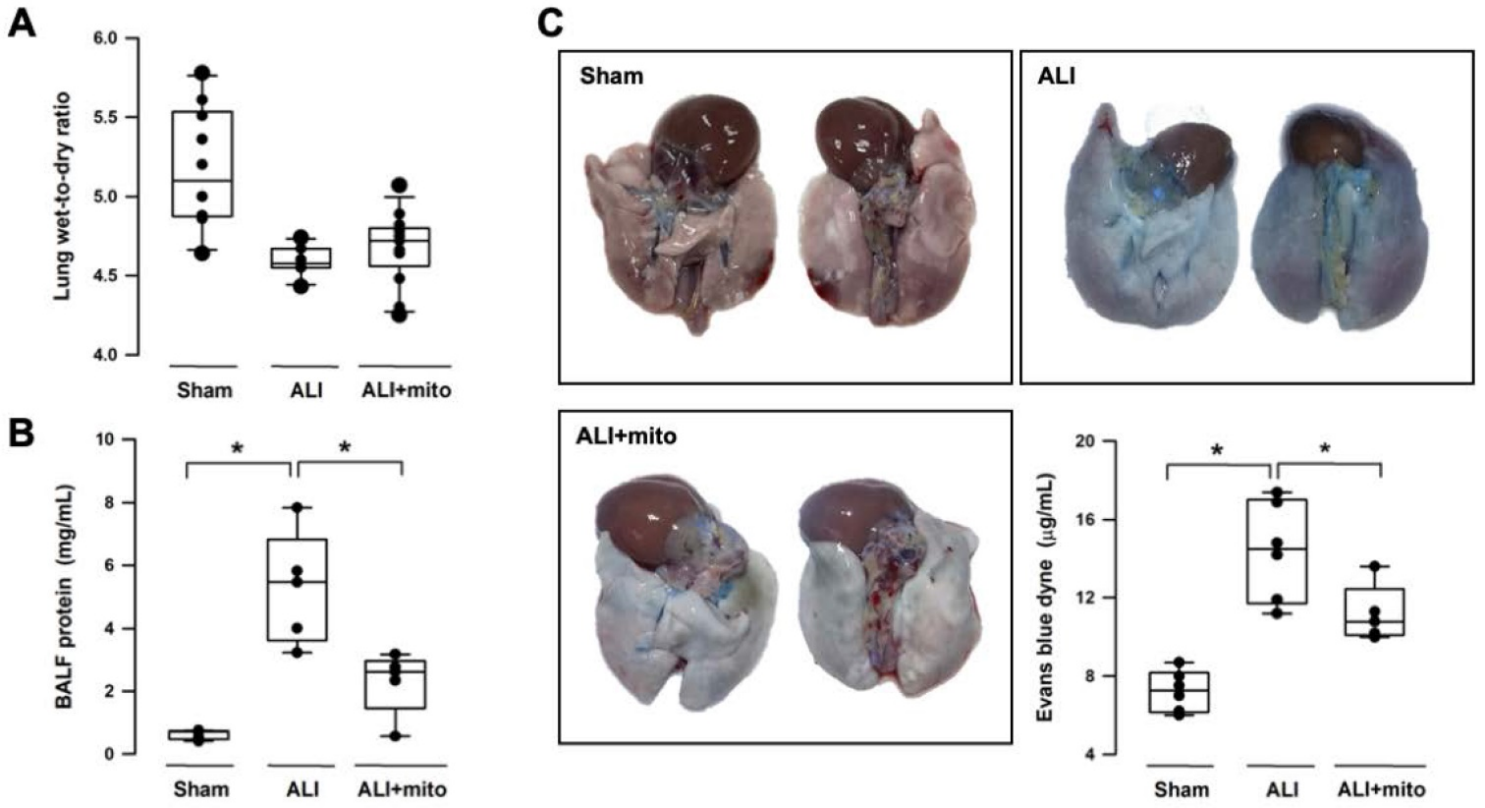
Changes in the alveolar-capillary permeability were assessed by degree of lung edema (lung wet-to-dry ratio, LWDR), protein content in the broncho-alveolar lavage fluid (BALF) and extravasated Evan blue dyne in the lung tissue. There were not differences in the LWDR among the three treatment groups **(A)**, but total protein discovered from the BALF **(B)** and Evan blue dyne **(C)** in the lung homogenates was significantly reduced in endotoxin-induced acute lung injury (ALI) rats treated with allogeneic mitochondria. Results are presented as box-and-whisker plots, in which the horizontal lines of color boxes indicate the 75^th^ percentile, median and 25^th^ percentile of the distribution, and the upper and lower whiskers indicate the maximal and minimal values. Data were analyzed using the Rank Sum Test, *P<0.05, n=5-12 different animals in each group.

Intratracheal LPS instillation successfully impaired pulmonary gas exchange, as shown by the significant reduction of PaO_2_ (95.9±5.0 mmHg vs 78.9±3.8 mmHg, sham vs ALI, respectively; P<0.001) and increase in PaCO_2_ (51.6±3.7 mmHg vs 61.1±3.8 mmHg, sham vs ALI, respectively; P<0.001) (Table 1). Level of HCO_3_^-^ was increased in the ALI group but there was no difference in arterial pH (Table 1). Following two doses of mitochondrial transplants, arterial O_2_ and CO_2_ partial pressures were improved in rats with ALI (Table 1). Saturation of oxygen content (SaO_2_) in arterial blood also improved significantly in the mitochondrial group (Table 1). Three different means were used to measure the integrity of the alveolar-capillary barrier in rats with ALI. Compared to shams, the LWDR was reduced in the lungs of ALI animals (Figure 2A) and mitochondria transplantation did not change the lung-weight ratio (Figure 2A). The BALF protein content and extravasated Evans blue dye content significantly decreased in the lung tissues of mitochondrial-transplanted animals to levels similar to the sham-operated group (Figures 2B and 2C), suggesting improvement in pulmonary endothelial permeability following mitochondrial transplantation.

### Transplantation of mitochondria potentiates pulmonary vascular endothelial function

**Figure 3:**
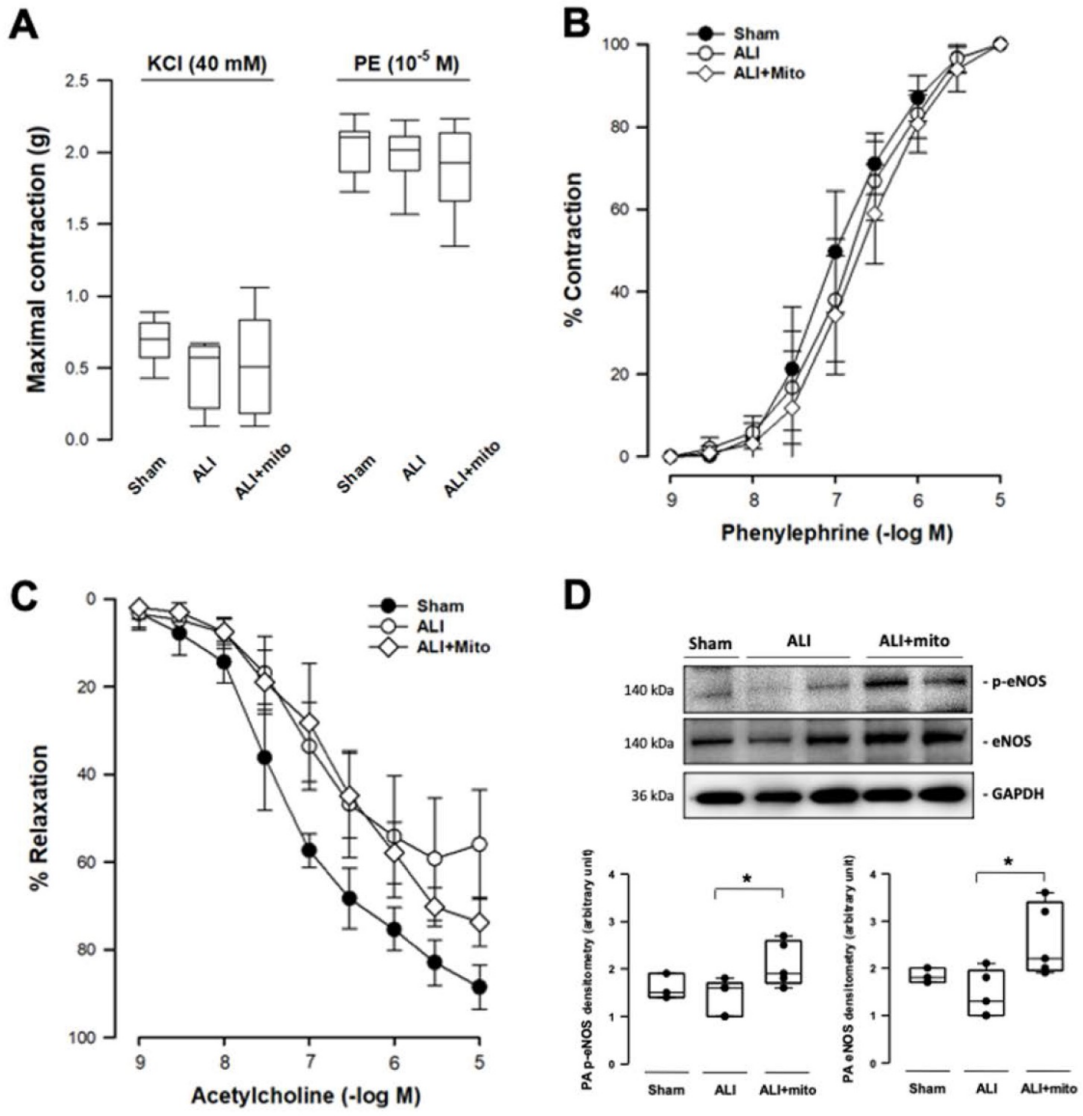
Measurements of isometric force of isolated pulmonary artery. **(A)** Contraction responses to KCl (40 mM) and maximal concentration of phenylephrine (PE, 10^-5^ M). **(B)** Contraction responses to cumulative addition of phenylephrine (PE, 10^-9^ to 10^-5^ M). There were no differences in the contraction responses to KCl depolarization and 1-adrenergic stimulation in the pulmonary artery segments of acute lung injury (ALI) rats with or without mitochondrial transplantation. **(C)** Endothelium-dependent relaxation response to cumulative addition of acetylcholine (10^-9^ to 10^-5^ M). The concentration response curves were significantly impaired in ALI groups, as compared with sham-operated animals. The maximal tension of relaxation induced by 10^-5^ M acetylcholine was significantly restored in the mitochondrial transplanted group (55.9±12.0 vs 73.6±5.6%, ALI vs mitochondrial groups, respectively; P=0.006 analyzed using the Rank Sum Test). n=6-8 different animals in each group for isometric force measurement. **(D)** Protein expressions of eNOS and phosphorylated eNOS (p-eNOS) in the rat pulmonary arteries. Results are presented as box-and-whisker plots, in which the horizontal lines of color boxes indicate the 75^th^ percentile, median and 25^th^ percentile of the distribution, and the upper and lower whiskers indicate the maximal and minimal values. Data were analyzed using the Rank Sum Test, *P<0.05, n=3-5 different animals in each group for Western blot analysis.

Transplantation of mitochondria did not affect the contraction responses to KCl depolarization and α1-adrenergic stimulation in the isolated pulmonary artery of rats with ALI (Figures 3A and 3B). However, the maximal tension of relaxation induced by 10^-5^ M acetylcholine restored significantly in the mitochondrial transplanted group (55.9±12.0 vs 73.6±5.6%, ALI vs mitochondrial groups, respectively; P=0.006; Figure 3C). However, the concentration-response curves of endothelial-dependent relaxation of pulmonary arteries were superimposed in the two ALI groups (Figure 3C). eNOS is one of the most important regulators in maintaining normal cellular function in the vascular endothelium 21. Protein expression of eNOS and its phosphorylated form (p-eNOS-S1177) in the pulmonary artery were suppressed in rats with ALI, but normalized following mitochondrial administration (Figure 3D).

### Transplantation of mitochondria attenuates damage of lung parenchyma

**Figure 4:**
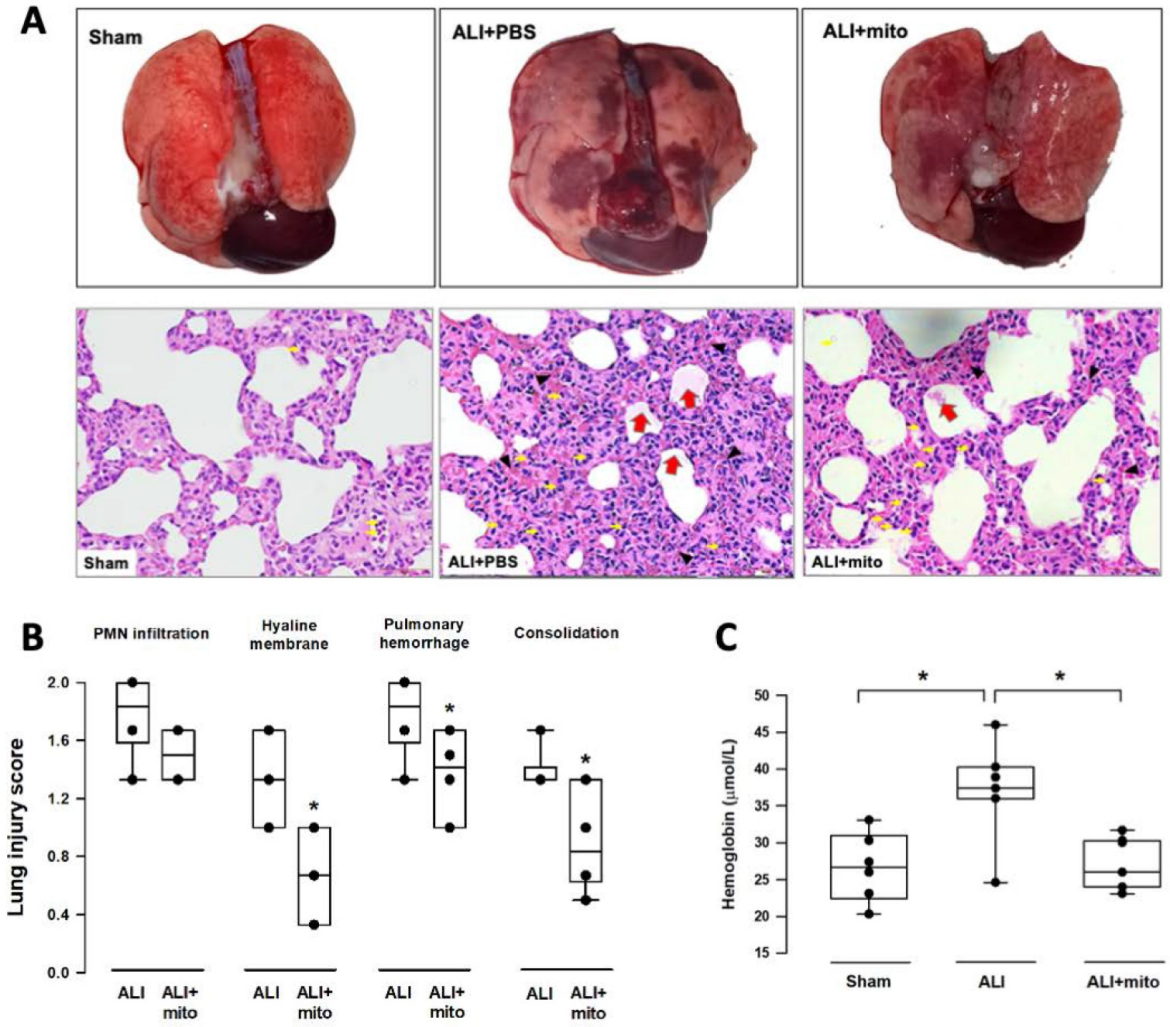
** (A)** Gross findings in lungs with acute lung injury (ALI) were heterogeneous, consisted of patchy consolidation and hemorrhage on the pleural surface of the lungs. The injured lungs were in unevenly bluish-red color, as compared with the normal pink aerated appearance in the lungs of sham-operated rats. The gross findings of lung lobes are presented as dorsal views. Representative lung tissue sections stained with hematoxylin and eosin show alveolar wall thickening and consolidation, neutrophilic infiltration in the alveolar and the interstitial spaces (yellow arrows), extravasation of erythrocytes in the lung interstitium (black arrowheads) and filling of proteinaceous debris or formation of hyaline membrane in the airspaces (red arrows) in the rats with ALI. **(B)** Severity of lung injury was scored using a semiquantitative histopathology score system 20, which evaluates lung injury by degree of neutrophil infiltration, amount of hyaline membranes or proteinaceous debris, level of lung hemorrhage and extent of parenchymal consolidation. **(C)** Quantification of lung hemorrhage by measuring hemoglobin content in the lungs. Results are presented as box-and-whisker plots, in which the horizontal lines of color boxes indicate the 75^th^ percentile, median and 25^th^ percentile of the distribution, and the upper and lower whiskers indicate the maximal and minimal values. Data were analyzed using the Rank Sum Test, *P<0.05, n=6-7 different animals in each group.

**Figure 5:**
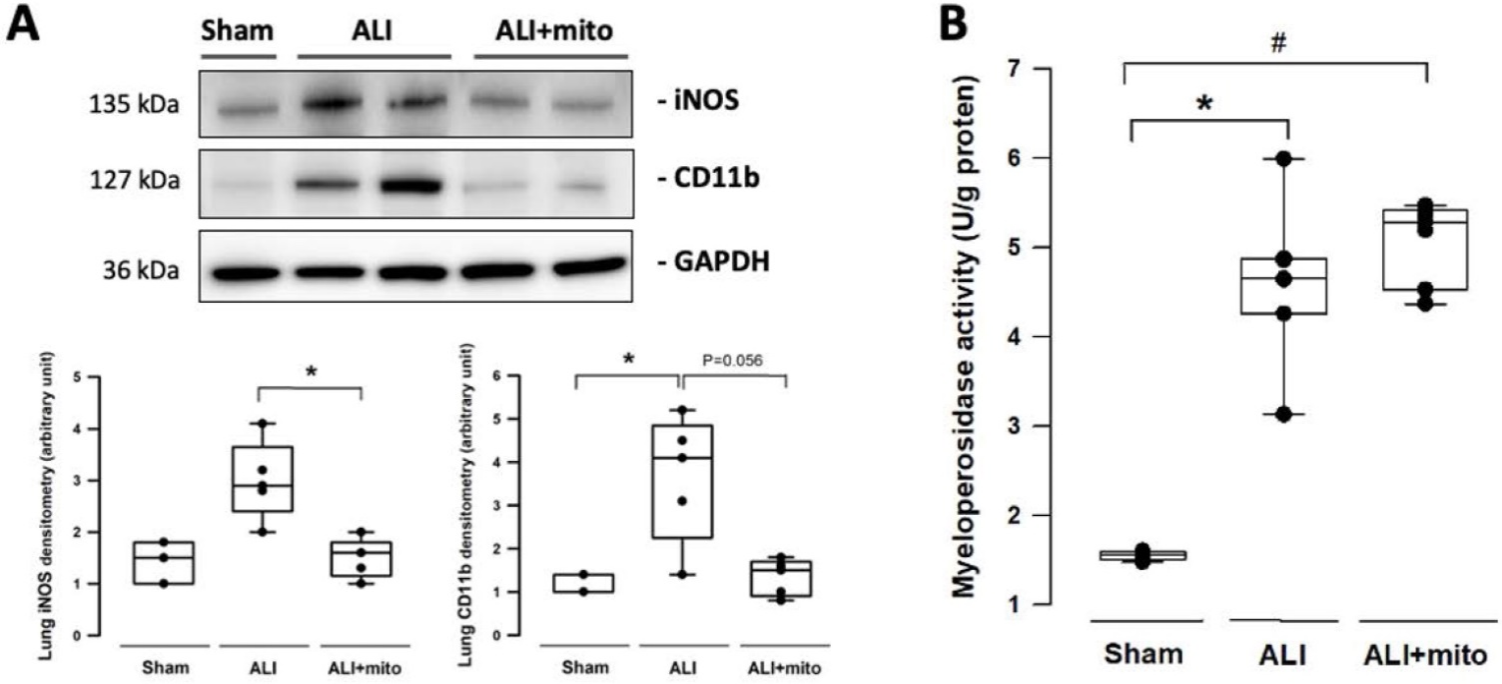
**(A)** Infiltration of leukocytes was assessed by expression of iNOS and CD11b in the lungs. **(B)** Measurement of myeloperoxidase activity in the lungs of rats with or without endotoxin-induced acute lung injury (ALI). Results are presented as box-and-whisker plots, in which the horizontal lines of color boxes indicate the 75^th^ percentile, median and 25^th^ percentile of the distribution, and the upper and lower whiskers indicate the maximal and minimal values. Data were analyzed using the Rank Sum Test, *P<0.05, n=3-7 different animals in each group.

The severity of lung damage was assessed using a lung injury score, pulmonary hemorrhage, and tissue inflammation. Lung injury was precipitated by increased neutrophil infiltration and red blood cell accumulation in the alveolar and interstitial spaces at 24 h after intratracheal lipopolysaccharide instillation (Figure 4A). Consolidation, alveolar ectasia, and hyaline membrane formation were noted in the lung parenchyma of rats with ALI (Figure 4A). Overall lung injury scores were significantly reduced in ALI animals received mitochondrial transplantation (1.57±0.11 vs 1.11±0.17, ALI vs ALI+mito, respectively; P=0.004 (Figure 4B). Tissue hemoglobin content was reduced in animals with mitochondria transplants compared to placebo rats with ALI, which indicates improvement in pulmonary hemorrhage in ALI (Figure 4C). Inflammatory cell infiltration was determined by the protein levels of iNOS and CD11b in the lung homogenates 17. Mitochondrial transplantation significantly suppressed expressions of iNOS and CD11b in the lungs of endotoxin-induced ALI (Figure 5A). However, no differences in the lung myeloperoxidase activity were found between ALI animals that received placebo or mitochondrial treatment (Figure 5B).

## Discussion

Dr. Mc Cully and his colleagues reported the first clinical application of mitochondrial transplantation in pediatric patients with ischemia-reperfusion-associated myocardial dysfunction 16. Mitochondria transplantation have since been considered as a revolutionary approach for regenerative medicine 22. Mitochondrial transplantation has the advantage of rapid isolation to meet clinical needs and studies also suggest that mitochondria are unlikely to induce alloreactivity and damage-associated molecular pattern molecules reaction 23. Therefore, mitochondria have potential for both syngeneic and allogeneic transplants 24. Furthermore, exogenous mitochondria can be conveniently administrated intravenously 14, 18.

Intratracheal lipopolysaccharide instillation destroys lung parenchyma through the generation of proteases and reactive oxygen and nitrogen species, which are produced by activated polymorphonuclear cells in the interstitial and alveolar compartments. This is a process that is comparable to the pathophysiological changes found in patients with ALI/ARDS 25. Previous studies found that intra-alveolar inflammatory reactions composes of a neutrophilic exudate in the initial 6 to 12 h, a monocytic exudate peaking at 24 h, followed by a lymphocytic exudate 26. After lipopolysaccharide instillation, we detected hypoxemia and hypercapnia, indicating impaired gas exchange due to development of alveolar dead space and ventilation-to-perfusion mismatch in these animals 27. The alveolar-capillary barrier is organized by a network of collagen and laminin that separates the epithelium and endothelium 28. The capillary endothelium is a semipermeable barrier for fluid exchange, whereas the alveolar epithelium is a tight layer that restricts the passage of water, electrolytes and hydrophilic solutes to the air space 29. Increased protein contents in the BALF, pulmonary hemorrhage, inflammatory cell infiltration, and parenchymal consolidation further confirmed alveolar-capillary disruption.

Under IVIS, we detected that the engraftment of transplanted mitochondria into the pulmonary circulation was significantly more enhanced in lungs of rats with ALI. The uptake of these exogenous mitochondria was further confirmed by the increased ATP concentrations in the lungs. Consistent with our previous report 18, these findings suggest that intravenous delivery of exogenous mitochondria can selectively locate and be taken up by areas of the pulmonary arterial system with mitochondrial dysfunction. After two mitochondrial transplants, the protein content recovered in BALF and extravasated Evans blue dye was significantly reduced, suggesting improved integrity of the monolayer capillary endothelium in preventing leakage of plasma proteins and albumin into the air-space 19, 30. However, lung water content measured by LWDR did not differ significantly among the three treatment groups. We speculate that the development of extensive alveolar ectasia, lung consolidation, and predominant infiltration of lymphocytes at 24 h after ALI 31 may have obscured the formation of pulmonary edema.

Pulmonary endothelial function was also assessed using vasomotor function tests and through the expression of eNOS in the pulmonary artery. The isometric tension analysis found no differences in the concentration response curves of phenylephrine and acetylcholine of ALI animals treated with PBS or mitochondria. However, the relaxation response to maximal concentrations of acetylcholine (endothelial-dependent relaxation induced by stimulating the muscarinic receptors) was significantly better in animals who received mitochondrial treatment. The increased isometric tension in the pulmonary arteries of ALI rats that received PBS at high acetylcholine concentrations might be due to a vasoconstriction reaction mediated by direct stimulation of the muscarinic receptors on the vascular smooth muscle cells where the endothelial layer is damaged or denuded 5, 32. Since the lung injury primarily originated from the bronchoalveolar site in this model, it was therefore reasonable that fewer changes in the vasoreactivity tests were detected in comparison to experimental models of direct pulmonary endothelial injury, such as through intravenous administration of oleic acid 5. Endothelial NOS is an important enzyme in endothelial cells that synthesizes the optimal amount of NO in order to maintain normal endothelial homeostasis 21, 33. Phosphorylation of eNOS at Ser1177 through Akt/Protein kinase B or AMP-activated protein kinase is a critical requirement for eNOS activation 34. The Western blot analysis confirmed that mitochondria transplantation restored eNOS and p-eNOS-S1177 levels in the pulmonary artery, providing further evidence that engraftment of exogenous mitochondria into the pulmonary circulation can improve endothelial function in rats with ALI. In addition, the increase in capillary endothelium integrity also reduces trans-endothelial migration of immune cells towards the inflammatory cascade in lung tissues 35. iNOS and CD11b are considered as cell markers of activated M1 macrophages during the pro-inflammatory phase of ALI 36. The significant suppression of iNOS and CD11b expression in injured lungs following mitochondrial transplantation imply decreased pro-inflammatory cell infiltration in rats with ALI.

The exact mechanisms underlying mitochondrial transplantation in tissue regeneration remains unclear, but three potential mechanisms have been proposed 37. The Ca^2+^ buffering capacity of mitochondria may attenuate the environmental Ca^2+^ overload during cellular stress through the opening of voltage-dependent anion channels 38. The second theory proposes that the internalization of exogenous mitochondria in recipient cells improves the mitochondrial function of target cells by increasing ATP generation and mitochondrial oxygen consumption 39. Finally, the viable exogenous mitochondria may release ATP into the extracellular environment and salvage the dysfunctional cells 37, 40. However, there is currently insufficient evidence to support the direct anti-inflammatory potential of transplanted mitochondria during tissue injury. It is then expected that we did not find any significant effects of mitochondrial transplant on the myeloperoxidase activity assay in the lung tissue exposed to endotoxin instillation.

There are a number of limitations in our study. First, the intracellular localization of these exogenous mitochondria was not determined in this study. Secondly, this study recorded very few ALI-related mortality throughout the study period. The effects of mitochondrial transplantation on other outcome measurements (e.g. overall mortality rate and lung parenchymal repair) were not studied. Thirdly, the optimal dose of mitochondria and the frequency of treatment were rather arbitrary, as there is still no general consensus on the standard dosing for mitochondrial transplantation 41. Fourthly, this was a pre-clinical, proof-of-concept research for the potential application of mitochondrial transplantation in ALI/ARDS and more mechanistic analysis are still under investigation in our laboratory.

**Figure 6:**
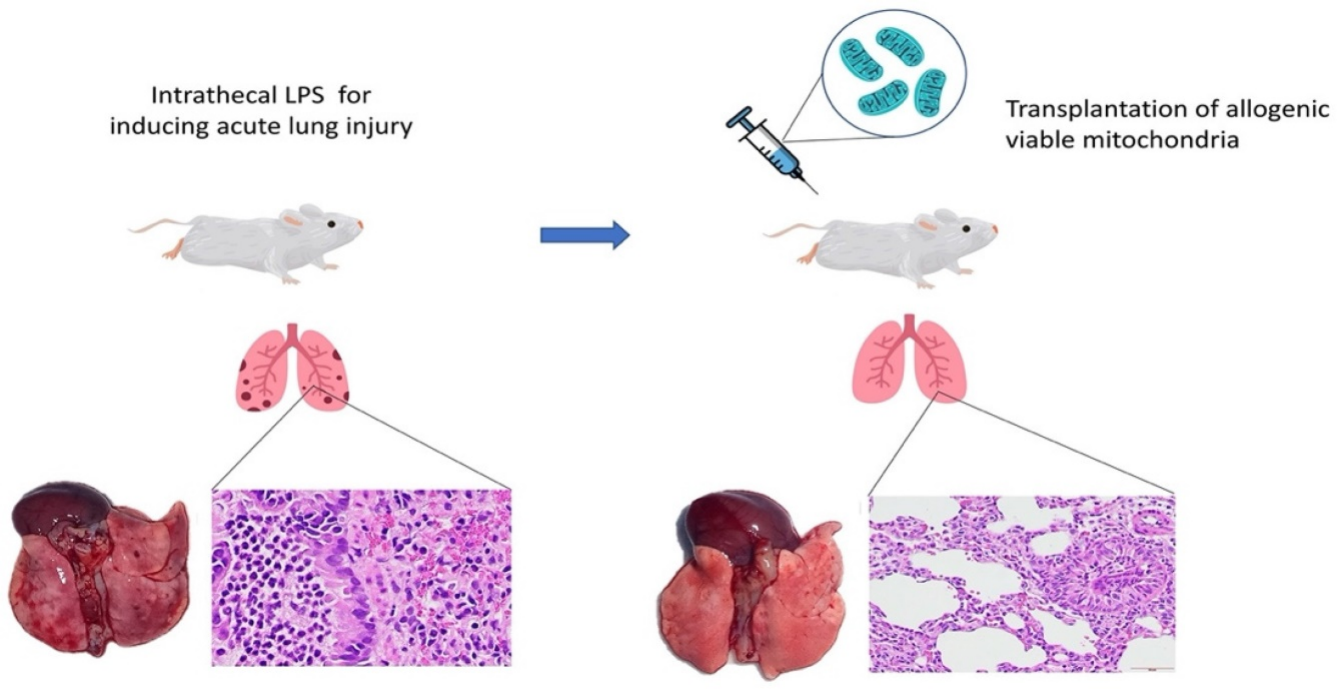
Graphic abstract of the study.

In conclusion, this is the first report demonstrating that intravenous transplantation of viable allogeneic mitochondria in rats with endotoxin-induced ALI significantly improves gas exchange by reducing alveolar-capillary barrier endothelial disruption (Figure 6). However, the effects on survival outcomes and long term recovery of pulmonary function in subjects with ALI after mitochondrial transplants require further investigation.

## Figures and Tables

**Table 1:** Measurements of arterial blood gases (ABG)

Parameters	Sham (n=10)	ALI (n=8)	ALI+Mito (n=8)
PaO_2_ (mmHg)	95.96±5.03	78.88±3.82*^,†^	93.44±5.29
PaCO_2_ (mmHg)	51.59±3.71	61.16±3.82*^,†^	56.11±4.54
SaO_2_ (%)	96.80±0.52	94.95±1.30*^,†^	96.50±0.93
HCO_3_^-^ (mmol/L)	28.38±2.58	32.84±3.07*	30.59±2.24
pH	7.363±0.03	7.342±0.03	7.345±0.03

Blood samples were collected via femoral artery and analyzed for ABG at 24 h after induction of acute lung injury (ALI); ALI+mito: rats with ALI received allogeneic mitochondrial transplantation; PaO_2_ and PaCO_2_: partial pressures of oxygen and carbon dioxide in artery blood; SaO_2_: oxygen saturation in the artery blood. Data were analyzed using the Mann-Whitney U test and are presented as mean±SD. *P<0.005 compared with sham group and ^†^P<0.05 compared with ALI+mito group.

## References

[1] Bellani G, Laffey JG, Pham T, Fan E, Brochard L, Esteban A (2016). Epidemiology, Patterns of Care, and Mortality for Patients With Acute Respiratory Distress Syndrome in Intensive Care Units in 50 Countries. Jama.

[2] Herold S, Gabrielli NM, Vadász I (2013). Novel concepts of acute lung injury and alveolar-capillary barrier dysfunction. Am J Physiol Lung Cell Mol Physiol.

[3] Shaver CM, Grove BS, Putz ND, Clune JK, Lawson WE, Carnahan RH (2015). Regulation of alveolar procoagulant activity and permeability in direct acute lung injury by lung epithelial tissue factor. Am J Respir Cell Mol Biol.

[4] Thompson BT, Chambers RC, Liu KD (2017). Acute Respiratory Distress Syndrome. N Engl J Med.

[5] Lam CF, Liu YC, Hsu JK, Yeh PA, Su TY, Huang CC (2008). Autologous transplantation of endothelial progenitor cells attenuates acute lung injury in rabbits. Anesthesiology.

[6] Huertas A, Guignabert C, Barberà JA, Bärtsch P, Bhattacharya J, Bhattacharya S (2018). Pulmonary vascular endothelium: the orchestra conductor in respiratory diseases: Highlights from basic research to therapy. Eur Respir J.

[7] Millar FR, Summers C, Griffiths MJ, Toshner MR, Proudfoot AG (2016). The pulmonary endothelium in acute respiratory distress syndrome: insights and therapeutic opportunities. Thorax.

[8] Kluge MA, Fetterman JL, Vita JA (2013). Mitochondria and endothelial function. Circ Res.

[9] Pacheu-Grau D, Rucktäschel R, Deckers M (2018). Mitochondrial dysfunction and its role in tissue-specific cellular stress. Cell Stress.

[10] Cloonan SM, Choi AM (2016). Mitochondria in lung disease. J Clin Invest.

[11] Supinski GS, Schroder EA, Callahan LA (2020). Mitochondria and Critical Illness. Chest.

[12] Lee YL, Obiako B, Gorodnya OM, Ruchko MV, Kuck JL, Pastukh VM (2017). Mitochondrial DNA Damage Initiates Acute Lung Injury and Multi-Organ System Failure Evoked in Rats by Intra-Tracheal Pseudomonas Aeruginosa. Shock.

[13] Hayakawa K, Esposito E, Wang X, Terasaki Y, Liu Y, Xing C (2016). Transfer mitochondria from astrocytes to neurons after stroke. Nature.

[14] Fang SY, Roan JN, Lee JS, Chiu MH, Lin MW, Liu CC (2021). Transplantation of viable mitochondria attenuates neurologic injury after spinal cord ischemia. J Thorac Cardiovasc Surg.

[15] Hayashida K, Takegawa R, Shoaib M, Aoki T, Choudhary RC, Kuschner CE (2021). Mitochondrial transplantation therapy for ischemia reperfusion injury: a systematic review of animal and human studies. J Transl Med.

[16] Emani SM, Piekarski BL, Harrild D, Del Nido PJ, McCully JD (2017). Autologous mitochondrial transplantation for dysfunction after ischemia-reperfusion injury. J Thorac Cardiovasc Surg.

[17] Pang YL, Chen BS, Li SP, Huang CC, Chang SW, Lam CF (2012). The preconditioning pulmonary protective effect of volatile isoflurane in acute lung injury is mediated by activation of endogenous iNOS. J Anesth.

[18] Hsu CH, Roan JN, Fang SY, Chiu MH, Cheng TT, Huang CC (2020). Transplantation of viable mitochondria improves right ventricular performance and pulmonary artery remodeling in rats with pulmonary arterial hypertension. J Thorac Cardiovasc Surg.

[19] Lam CF, Roan JN, Lee CH, Chang PJ, Huang CC, Liu YC (2011). Transplantation of endothelial progenitor cells improves pulmonary endothelial function and gas exchange in rabbits with endotoxin-induced acute lung injury. Anesth Analg.

[20] Patel BV, Wilson MR, Takata M (2012). Resolution of acute lung injury and inflammation: a translational mouse model. Eur Respir J.

[21] Heiss C, Rodriguez-Mateos A, Kelm M (2015). Central role of eNOS in the maintenance of endothelial homeostasis. Antioxid Redox Signal.

[22] Roushandeh AM, Kuwahara Y, Roudkenar MH (2019). Mitochondrial transplantation as a potential and novel master key for treatment of various incurable diseases. Cytotechnology.

[23] Preble JM, Pacak CA, Kondo H, MacKay AA, Cowan DB, McCully JD Rapid isolation and purification of mitochondria for transplantation by tissue dissociation and differential filtration. J Vis Exp. 2014: e51682.

[24] Ramirez-Barbieri G, Moskowitzova K, Shin B, Blitzer D, Orfany A, Guariento A (2019). Alloreactivity and allorecognition of syngeneic and allogeneic mitochondria. Mitochondrion.

[25] Rittirsch D, Flierl MA, Day DE, Nadeau BA, McGuire SR, Hoesel LM (2008). Acute lung injury induced by lipopolysaccharide is independent of complement activation. J Immunol.

[26] Ulich TR, Watson LR, Yin SM, Guo KZ, Wang P, Thang H (1991). The intratracheal administration of endotoxin and cytokines. I. Characterization of LPS-induced IL-1 and TNF mRNA expression and the LPS-, IL-1-, and TNF-induced inflammatory infiltrate. Am J Pathol.

[27] Wagner PD (2015). The physiological basis of pulmonary gas exchange: implications for clinical interpretation of arterial blood gases. Eur Respir J.

[28] D'Agnillo F, Zhang X, Williams MC (2020). Structural Integrity of the Alveolar-Capillary Barrier in Cynomolgus Monkeys Challenged with Fully Virulent and Toxin-Deficient Strains of Bacillus anthracis. Am J Pathol.

[29] Herrero R, Sanchez G, Lorente JA (2018). New insights into the mechanisms of pulmonary edema in acute lung injury. Ann Transl Med.

[30] Schumacher J, Binkowski K, Dendorfer A, Klotz KF (2003). Organ-specific extravasation of albumin-bound Evans blue during nonresuscitated hemorrhagic shock in rats. Shock.

[31] Li H, Du S, Yang L, Chen Y, Huang W, Zhang R (2009). Rapid pulmonary fibrosis induced by acute lung injury via a lipopolysaccharide three-hit regimen. Innate Immun.

[32] Ludmer PL, Selwyn AP, Shook TL, Wayne RR, Mudge GH, Alexander RW (1986). Paradoxical vasoconstriction induced by acetylcholine in atherosclerotic coronary arteries. N Engl J Med.

[33] Förstermann U, Münzel T (2006). Endothelial nitric oxide synthase in vascular disease: from marvel to menace. Circulation.

[34] Chen CA, Druhan LJ, Varadharaj S, Chen YR, Zweier JL (2008). Phosphorylation of endothelial nitric-oxide synthase regulates superoxide generation from the enzyme. J Biol Chem.

[35] Reglero-Real N, García-Weber D, Millán J (2016). Cellular Barriers after Extravasation: Leukocyte Interactions with Polarized Epithelia in the Inflamed Tissue. Mediators Inflamm.

[36] Johnston LK, Rims CR, Gill SE, McGuire JK, Manicone AM (2012). Pulmonary macrophage subpopulations in the induction and resolution of acute lung injury. Am J Respir Cell Mol Biol.

[37] Bertero E, Maack C, O'Rourke B (2018). Mitochondrial transplantation in humans: “magical” cure or cause for concern?. J Clin Invest.

[38] Romero-Garcia S, Prado-Garcia H (2019). Mitochondrial calcium: Transport and modulation of cellular processes in homeostasis and cancer (Review). Int J Oncol.

[39] Pacak CA, Preble JM, Kondo H, Seibel P, Levitsky S, Del Nido PJ (2015). Actin-dependent mitochondrial internalization in cardiomyocytes: evidence for rescue of mitochondrial function. Biol Open.

[40] Masuzawa A, Black KM, Pacak CA, Ericsson M, Barnett RJ, Drumm C (2013). Transplantation of autologously derived mitochondria protects the heart from ischemia-reperfusion injury. Am J Physiol Heart Circ Physiol.

[41] McCully JD, Levitsky S, Del Nido PJ, Cowan DB (2016). Mitochondrial transplantation for therapeutic use. Clin Transl Med.

